# 

**Published:** 1882-07

**Authors:** 


| | PAGE |
|---|---|
| **ORIGINAL COMMUNICATIONS:** | |
| The Deliverances of the Retina. By Geo. E. Blackham, M. D., F. R. M. S., Dunkirk, N. Y., | 529 |
| "Sewer Gas" and its Dangers. By A. R. Davidson, M. D., | 537 |
| **CLINICAL REPORTS:** | |
| Case of Imperforate Anus. Reported by Herman Mynter, M. D., | 550 |
| Aortic Aneurism—Report of a Case Measuring Five by Eight Inches in Diameter and Weighing Five Pounds. By Chas. C. F. Gay, M. D., Buffalo, N. Y., | 552 |
| A Case of Typhoid Fever with a Peculiar Complication. Reported by J. H. Pryor, M. D., Resident Physician General Hospital, | 555 |
| **SOCIETY REPORTS:** | |
| Buffalo Medical and Surgical Association, | 556 |
| The New York Code, | 559 |
| **EDITORIAL.** | |
| The American Medical Association, | 561 |
| Editorial Notes, | 568 |

